# Affimer-Based Europium Chelates Allow Sensitive Optical Biosensing in a Range of Human Disease Biomarkers

**DOI:** 10.3390/s21030831

**Published:** 2021-01-27

**Authors:** Eiman Al-Enezi, Alexandre Vakurov, Amy Eades, Mingyu Ding, Gin Jose, Sikha Saha, Paul Millner

**Affiliations:** 1 Bionanotechnology Group, School of Biomedical Science, University of Leeds, Leeds LS2 9JT, UK; eman.a.s.85@hotmail.com (E.A.-E.); A.V.Vakourov@leeds.ac.uk (A.V.); amy_eades@yahoo.co.uk (A.E.); ddmmyy0521@gmail.com (M.D.); 2 School of Chemical and Process Engineering, University of Leeds, Leeds LS2 9JT, UK; G.Jose@leeds.ac.uk; 3 School of Medicine, University of Leeds, Leeds LS2 9JT, UK; s.saha@leeds.ac.uk

**Keywords:** biosensor, lanthanide, europium chelates, Affimer, biomarkers

## Abstract

The protein biomarker measurement has been well-established using ELISA (enzyme-linked immunosorbent assay), which offers good sensitivity and specificity, but remains slow and expensive. Certain clinical conditions, where rapid measurement or immediate confirmation of a biomarker is paramount for treatment, necessitate more rapid analysis. Biosensors offer the prospect of reagent-less, processing-free measurements at the patient’s bedside. Here, we report a platform for biosensing based on chelated Eu^3+^ against a range of proteins including biomarkers of cardiac injury (human myoglobin), stroke (glial fibrillary acidic protein (GFAP)), inflammation (C-reactive protein (CRP)) and colorectal cancer (carcinoembryonic antigen (CEA)). The Eu^3+^ ions are chelated by modified synthetic binding proteins (Affimers), which offer an alternative targeting strategy to existing antibodies. The fluorescence characteristics of the Eu^3+^ complex with modified Affimers against human myoglobin, GFAP, CRP and CEA were measured in human serum using λ_ex_ = 395 nm, λ_em_ = 590 and 615 nm. The Eu^3+^-Affimer based complex allowed sensitive detection of human myoglobin, GFAP, CRP and CEA proteins as low as 100 fM in (100-fold) diluted human serum samples. The unique dependence on Eu^3+^ fluorescence in the visible region (590 and 615 nm) was exploited in this study to allow rapid measurement of the analyte concentration, with measurements in 2 to 3 min. These data demonstrate that the Affimer based Eu^3+^ complexes can function as nanobiosensors with potential analytical and diagnostic applications.

## 1. Introduction

Over the past few decades, trivalent lanthanides complexes have attracted much attention in the field of fluorescent labels and sensors due to several desirable features. They are characterised by excitation wavelengths <400 nm whilst emission is at 545–700 nm. Self-quenching and photobleaching is absent when conjugated to other molecules [1]. Their photochemical characteristics in the form of high quantum yield, sharp emission peaks and large Stoke’s shift are all favorable features for biological labels [2,3,4,5,6]. During the excited state, lanthanide life times are significantly long (µs to ms) which aids in eliminating the common problem of background auto-fluorescence in complex media [7,8].

**Figure 1:**
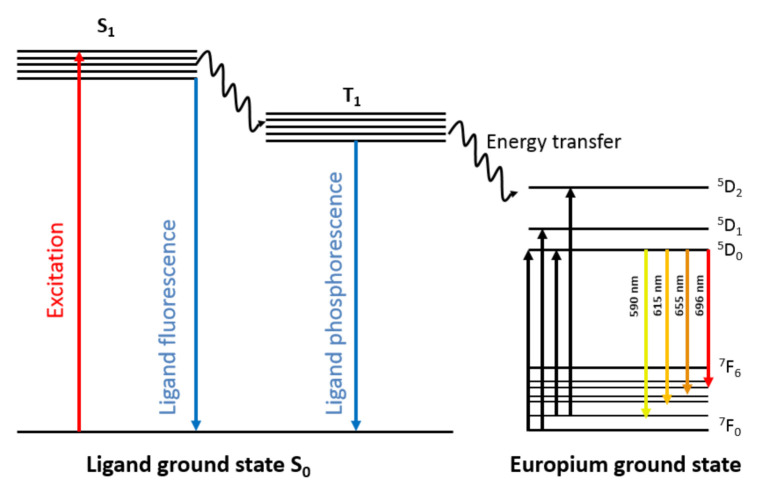
Schematic representation of the fluorescence emission processes occurring in responsive Eu^3+^ complex. The ligand absorbs the UV light and is excited from ground level S_0_ to S_1_ level after transfer of energy to the 5D_n_ level in the Eu^3+^. The ion emits fluorescence as a result of intramolecular energy transfer from 5D_0_ level to one of the lower energy 7F levels.

Lanthanides ions, when bound by chelators in general can absorb in the UV light region, allowing them to emit strong fluorescence in the visible region. The ideal ligand (chelator), therefore, must be efficient in ligand-to-metal energy transfer and possess aromatic group(s) that can act as an antennas to increase the UV light absorption [9,10,11]. Trivalent Eu^3+^ is a family member of the lanthanides which shows bright fluorescence when chelated by organic ligands. The Eu^3+^ complexes emit intense red fluorescence upon excitation with UV light [12]. The ligand (chelator) absorbs the excitation light via ligand π-π transition from the aromatic ring(s) and as a result the absorbed energy is transferred from the S1 level in the ligand to the 5D^n^ orbital in the Eu^3+^. Then, Eu^3+^ ions emit fluorescence when energy is transferred from the 5D_0_ orbital to one of the 7F levels as shown in Figure 1 [13]. 

The strongest emission peaks among several Eu^3+^ transition levels are between ^5^D_0_–^7^F_1_ and ^5^D_0_–^7^F_2_ at 590 nm, and 615 nm, respectively. The final F-F transition is mainly responsible for the long lifetime of the fluorescence, and it is fairly resistant to environmental factors. The unique fluorescence properties of the Eu^3+^ complexes offer many applications in the field of bioimaging, fluorescent probes, drug delivery, medical diagnosis and biosensors [14,15,16,17,18]. 

Biosensors overcome many limitations suffered by conventional analytical procedures which may be time consuming, are complex and need costly equipment [19,20,21,22,23]. Optical biosensors are considered as good alternatives to conventional analytical techniques to provide rapid, highly sensitive and real-time measurements [24,25]. Several attempts have been aimed at using antibody based biosensors for the above applications [14]. However, the limitations of the antibody-based systems prevented translation into clinical practice [26]. Despite the widespread applicability of antibodies as a research tools and therapeutic agents, they have several limitations [27,28,29]. Their complex structure, with light and heavy chains, leads to unstable domain association in the production of small Fv fragments [30]. Complicated cloning steps are required for the development of recombinant antibodies and the manufacture of antibodies is expensive and time-consuming. It also depends on use of live animals, which is undesirable [31]. 

An alternative solution is to use recently developed synthetic binding protein, Affimers [32,33,34,35]. The Affimers scaffold is derived from a consensus of a large number of plant cystatins, which are small protein inhibitors of cysteine proteases approximately 100 amino acids long. Affimers consist of a single α-helix and four antiparallel β-strands, lack cysteines and glycosylation sites and can be expressed in E. coli in large amounts. Affimers have two variable regions, each with nine randomised amino acids located between the first and second β strands (VR1) and between the third and fourth β strands (VR2) [26,35,36]. They are thermostable and some have melting temperatures up to 100 °C. This enables long-term storage at ambient temperature. In recent studies, Affimers have been used as a biorecognition elements for biosensing applications with promising results that can be built on, to develop a sensitive and cost-effective tool for rapid detection of proteins of interest [26,37,38,39]. In this study, we aimed to fabricate an Affimer based-optical biosensor using chelated Eu^3+^ to detect specific proteins.

## 2. Materials and Methods

### 2.1. Affimer Expression and Purification

Plasmids coding sequence for Affimers from the BioScreening Technology Group (BSTG), University of Leeds were used to express specific Affimer against human myoglobin and GFAP. *PET11 (a)* vectors containing specific Affimers coding sequence were transformed into BL21 Star™ (DE3) *E. coli* by a heat shock. Cells were allowed to grow at 37 °C and 230 rpm shaking overnight. The cultures in LB medium, supplemented with 100 µg/mL carbenicillin were induced with 0.1 mM isopropyl β-d-1-thiogalactopyranoside (IPTG). Cells were then harvested and lysed in 1 mL lysis buffer (300 mM NaCl, 50 mM NaH_2_PO_4_, 30 mM imidazole, 10% (*v*/*v*) glycerol, pH 7.4). Ni^2+^-NTA chelate chromatography was used to purify the Affimers which bear a His_6_ C-terminal tag. Affimers were eluted using elution buffer (500 mM NaCl, 50 mM NaH_2_PO_4_ and 300 mM imidazole, 20% (*v*/*v*) glycerol, pH 7.4), and a Nanodrop spectrophotometer was used to measure the concentration of the purified Affimer at 280 nm.

### 2.2. Affimer Functionalisation

**Figure 2:**
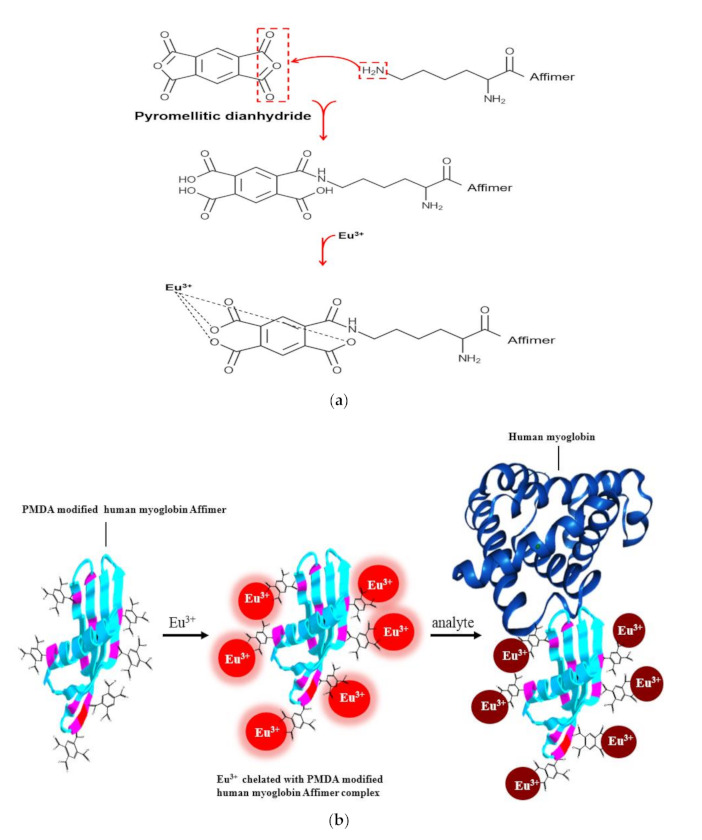
Schematic showing reaction of PMDA with lysine on the Affimer and the analyte binding. (**a**), Affimers were functionalised by conjugating PMDA to the scaffold. The modification forms a peptide bond between carboxylic groups on the PMDA to the Affimer lysines, whilst the other PMDA anhydride becomes hydrolyses, providing two additional carboxy groups. (**b**), the analyte (human myoglobin) binds to the Affimer variable loops and thereby partially shields the chelated Eu^3+^ from excitation.

Pyromellitic dianhydride (PMDA) at 1 M (218.12 mg/mL) was prepared and dissolved in 1 mL of dimethyl sulfoxide (DMSO) and then diluted to a final concentration of 100 mM (21.8 mg/mL). Then, 100 µL of 100 mM PMDA was then mixed with 1 mL of Affimer at 1 mg/mL and incubated for 2 h at 20 °C. The mixture was desalted using a Sephadex G-25, according to the manufacturer’s instructions. A schematic of the reaction of PMDA with the Affimer is shown in Figure 2a.

### 2.3. Fluorimeter Measurement of the Chelated Eu^3+^ to PMDA Modified Affimer

One hundred µM (15.2 mg/mL) of Eu(NO_3_)_3_ was prepared in 100 mM PBS then 1 mL of the solution was mixed with 20 µL of 50 µM (625 ng/mL) functionalised Affimer. The mixture was vortexed followed by immediate measurement of emission using a QEPro spectrometer (Ocean Optics) at 395 nm excitation. Analyte concentrations ranged from 100 fM to 100 nM were mixed with the Eu^3+^-Affimer complex and incubated for 5 min at room temperature. Then emission spectra of the complex were measured and recorded (Figure 2b). A KVANT 395 nm laser (395LM-120-FC-1V) was used for excitation at a current of 100 mA. Fluorescence emission data was recorded using Ocean software. Prior to measurement, the dark background current of the laser was subtracted and all measurements were made for 120 s for each sample. FEL0500 interference filter (Thorlabs) was placed in the emission path to cut off auto-fluorescence at λ < 500 nm. Data were exported into GraphPad Prism 7 for analysis.

### 2.4. Plate Reader Measurement of the Chelated Eu^3+^ to the PMDA Modified Affimer

Eu(NO_3_)_3_ at 100 µM was prepared in PBS (100 mM), then 200 µL of the prepared solution was added into each well of a glass 96-well plate (Zinsser Analytic, Eschborn, Germany). Subsequently, 20 µL of 10 µM (25 ng/mL) analyte was added to the first well and followed by 10-fold serial dilutions from 100 fM to 100 nM. Three replicates per well were made. Functionalised Affimer was added to a concentration of 1 µM (2.5 ng/mL) per well and incubated for 5 min at 25 °C, followed by measurement of emission run through a <500 nm emission peak cut-out filter in a BMG Fluostar plate reader. The plate reading start time was 20 µs after the exciting pulse and the integration time was adjusted to 50 s. Each replicate was read eight times per well at 25 °C. BMG Optima based software was used to read the signals from the 96-well plates in time-resolved fluorescence mode. Real-time data was also observed and recorded. Sandwich assays were also conducted using polyclonal antibodies to ascertain the size effect on the percentage change in fluorescence quenching of the Eu^3+^ complex. After adding 20 µL of 10 µM (25 ng/mL) analyte and incubating for 5 min at 25 °C, 20 μL of 1 μM of anti- CRP polyclonal antibody (Sigma Aldrich, St. Louis, MI, USA) at a concentration of 100 nM (3 ng/mL) was added to each well and further incubated for 5 min at room temp; followed by measurement of emission. Ethylenediaminetetraacetate (EDTA) and tri-sodium citrate stock solution were prepared in PBS at a concentration of 100 μM. After preparing the sandwich assay, 10 μM (2.92 μg/mL) EDTA or 1 μM (294.1 ng/mL) tri-sodium citrate were diluted 100× to a final concentration of 100 nM (29.2 ng/mL) and 10 nM (2.941 ng/mL), respectively into each well followed by immediate measurement of emission.

## 3. Results

### 3.1. Affimer Expression and Purification

The specificity of the Eu^3+^ complex was dependent upon the ability of the Affimer to bind to the target biomarker (human myoglobin, GFAP, CEA and CRP). Affimers against human biomarker proteins were selected and subcloned into a *pET11 (a)* vector for protein production. Affimers that contained lysine in the binding loops were excluded from further analysis in order to ensure that only lysines on the Affimer scaffold were modified by PMDA. A single cysteine residue was also introduced at the C-terminus of the Affimer scaffold to be used for other modifications. This site offers advantageous control of the orientation of Affimers when used in other detection and imaging assays. For example, others have shown successful conjugation of Affimer utilising cysteine in biosensors and targeted nanoparticles [34]. Our system did not require strict Affimer orientation because the Affimer-Eu^3+^ complex is essentially a homogenous liquid phase assay. However, when immobilisation of the bioreceptor is required, specific strategies are carefully considered to increase the bioreceptor immobilisation efficiency, which is heavily reliant on the bioreceptor orientation. Specialised site-specific orientation strategies are available for bioreceptors immobilisation [40]. Affimer binding specificity of all selected Affimers against human myoglobin, GFAP, CRP and CEA were checked prior to use in the project to ensure the successful outcome of the assay. Several approaches were used to confirm the selective binding between the bioreceptors (Affimer) and the protein of interest including ELISA, immunoprecipitation assays and SPR (surface plasmon resonance). The dissociation constant values (k_D_) of the Affimer-analyte binding affinity were ranged from pM-nM, which are in keeping with the k_D_ value of the antibody-antigen interaction [41].

### 3.2. Eu^3+^ Complex Excitation and Emission Spectra

**Figure 3:**
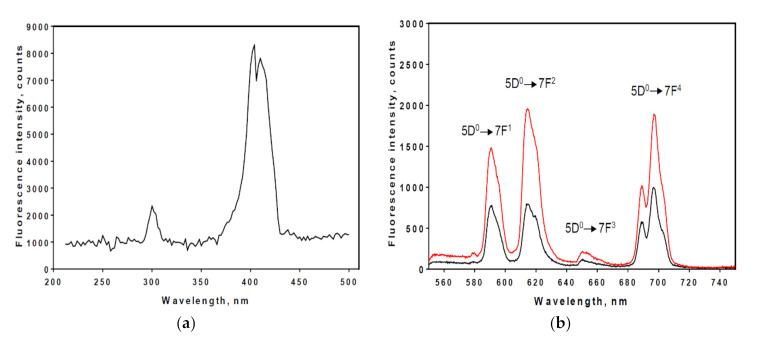
Excitation and emission spectra of Eu^3+^ complex. (**a**), peak excitation wavelengths for Eu^3+^ complex were measured and using λ_em_ = 615 nm; (**b**), peak emission wavelengths for Eu^3+^ complexes were measured for free Eu^3+^ (black line peaks) and Eu^3+^ chelated by PMDA modified anti-human myoglobin Affimer complex (red line peaks). The electronic transition corresponding to the emission peaks are indicated. λ_ex_ = 395 nm.

The excitation spectra of 100 µM Eu^3+^ complex in PBS is shown in Figure 3a. The excitation spectrum consists of two bands, which arise from the UV light absorption of the chelator (PMDA) and from direct F–F electron transitions of Eu^3+^. The most prominent excitation peak of the Eu^3+^ is at 395 nm which is caused by direct excitation of the 5L_6_ level of Eu^3+^. This excitation wavelength was then used for the measurement of fluorescence spectra of chelated Eu^3+^ with modified Affimer. Following excitation at 395 nm, the emission from Eu^3+^ chelated to PMDA modified anti-human myoglobin Affimers were recorded and was similar to that observed by other groups (Binnemans, 2015). The transition corresponding to their emission peaks are shown in Figure 3b. The strongest emission peaks were observed around 590 nm, 615 nm, and 696 nm, attributed to the ^5^D_0_→^7^F_1_, ^5^D_0_→^7^F_2_ and ^5^D_0_→^7^F_4_ transitions, respectively.

### 3.3. Fluorescence of Chelated Eu^3+^ to PMDA Modified Affimers Complexes by Analyte

**Figure 4:**
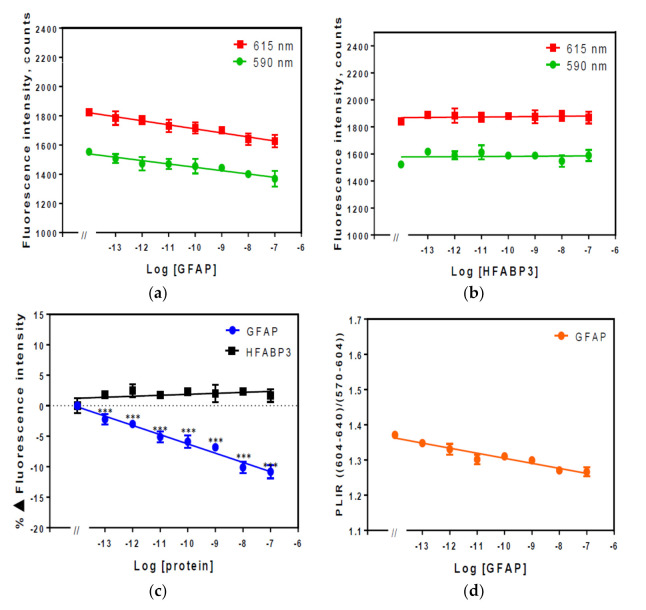
Fluorescence intensity spectra of Eu^3+^ chelated by PMDA modified anti-GFAP Affimer complex in 1% (*v*/*v*) serum. Fluorescence was measured at 590 and 615 nm with *λ_ex_* = 395 nm. (**a**) Fluorescence intensity spectra of Eu^3+^ chelated PMDA modified anti-GFAP Affimer; (**b**) fluorescence intensity spectra of the same complex after HFABP3 added; (**c**), percentage change in fluorescence intensity; (**d**) photoluminescence intensity ratio. Data are means ± SEM (*n* = 4). Some error bars are smaller than data points (*, ** and *** indicate significance with *p*-value < 0.05, 0.01, and 0.001 respectively of the specific analyte (GFAP) compared to the control (HFAPB3) data).

**Figure 5:**
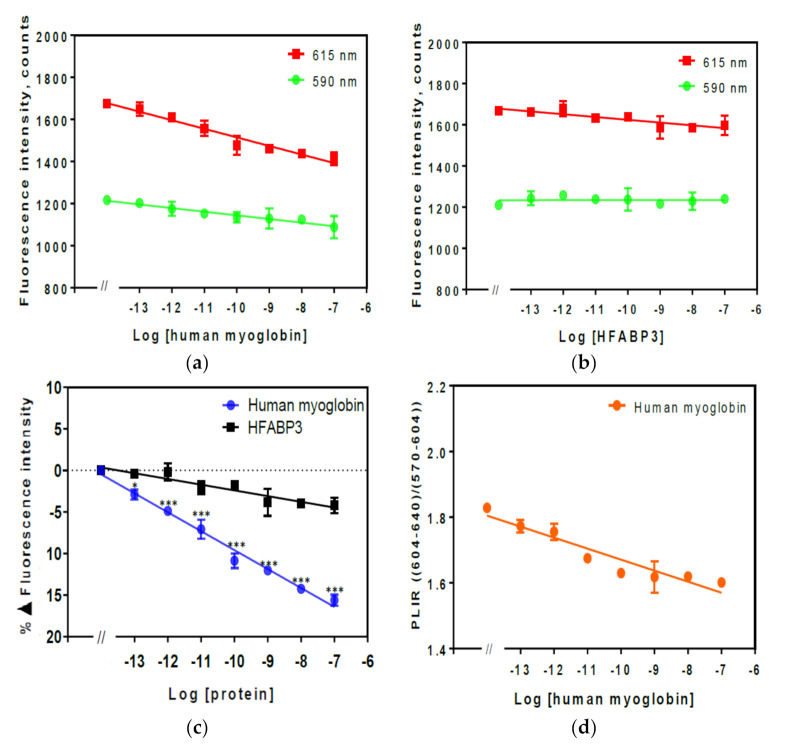
Fluorescence intensity spectra of Eu^3+^ chelated by PMDA modified anti-human myoglobin Affimer complex in 1% (*v*/*v*) human serum. Fluorescence was measured at 590 and 615 nm with *λ_ex_* = 395 nm. (**a**) Fluorescence intensity spectra of Eu^3+^ chelated PMDA modified anti-human myoglobin Affimer; (**b**), fluorescence intensity spectra of the same complex after HFABP3 (control) added; (**c**) percentage change in fluorescence intensity; (**d**) photoluminescence intensity ratio. Data are means ± SEM (n = 4). Some error bars are smaller than data points (*, ** and *** indicate significance with *p*-value < 0.05, 0.01, and 0.001 respectively of the specific analyte (human myoglobin) compared to the control (HFAPB3) data).

The Eu^3+^ complex was chelated by PMDA modified anti-GFAP and anti-human myoglobin Affimers and fluorescence intensity peaks were measured against different concentrations ranged from 100 fM–100 nM of their specific analytes in 1% (*v*/*v*) diluted human serum as shown in Figure 4 and Figure 5, respectively. The human serum concentration from 1–10% (*v*/*v*) were investigated by that beyond 1–2% (*v*/*v*) human serum, the quenching effects of the analyte was suppressed. 

The overall emission spectra of Eu^3+^ complex showed substantial quenching by increasing concentration of the analyte. The fluorescence peaks at 590 nm and 615 nm for the Eu^3+^ complex and their respective fluorescence quenching plots at different concentrations of GFAP are shown in Figure 4a. These findings suggest that when the Eu^3+^complex binds to the target GFAP protein (via the anti-GFAP Affimer), the fluorescence intensity decreases indicating that the Eu^3+^ complex was specific to the target antigen. This was further confirmed when human heart fatty acid binding protein (HFABP3) was used as a control against the same Eu^3+^ complex (Figure 4b). Here, the increased concentration of HFABP3 resulted in no obvious change in the fluorescence intensity of the Eu^3+^ complex. The percentage change in the fluorescence intensity comparing to the targeted Eu^3+^ complex (GFAP) versus the control (HFABP3) was quantified in a normalised data plot (Figure 4c) and the photoluminescence intensity ratio (PLIR) of the transitions ^5^D_0_→^7^F_2_ and ^5^D_0_→^7^F_1_ (Figure 4d). The normalised data showed a statistically significant change in fluorescence intensity of the targeted Eu^3+^ complex with a minimum of ~2.22 ± 0.85% difference at 100 fM (0.0055 pg/mL, *p* < 0.001) increasing to ~10.82 ± 1.08% at 100 nM (5.5 ng/mL, *p* < 0.001). 

The PLIR is defined by the ratio I(604–640 nm)/I(570–604 nm) which is the ratio of integrated intensity of electric dipole transition to magnetic dipole transition [42]. It provides information about the distortion from local sites inversion symmetry of the Eu^3+^ in the chelator complex. Figure 4d shows the PLIR values, which range from 1.38 to 1.25 nm with increasing concentration of the GFAP, suggesting a strong deformation of the symmetry around the Eu^3+^. This indicates that Eu^3+^ fluorescence changes that were observed are induced by changes within the Affimer modified with PMDA (ligand) complex and not the surrounding environment.

### 3.4. Fluorescence Intensity of Eu^3+^ Chelated to PMDA Modified Anti-Human Myoglobin Affimer Complex

The fluorescence spectrum of Eu^3+^ chelated to PMDA modified anti-human myoglobin complex was also investigated in a similar manner to Eu^3+^ chelated by PMDA modified anti-GFAP Affimer complex. The data are shown in Figure 5. The fluorescence intensity of the Eu^3+^ complex changed substantially with increased concentration of myoglobin. The fluorescence intensity peaks for the Eu^3+^ complex with different concentrations of myoglobin are shown in Figure 5a where fluorescence quenching can be observed with increased concentration of myoglobin. Similarly, no changes were observed in the fluorescence intensity of Eu^3+^ complex when the control analyte HFABP3 was added as shown in Figure 5b. The percentage change in fluorescence intensity in the Eu^3+^ chelated by PMDA modified anti- human myoglobin Affimer showed a similar pattern to what was observed in the GFAP complex with a ~2.88 ± 0.59% difference observed at a concentration of 1 pM (0.015 pg/mL) of the analyte (*p* < 0.05) and ~15.60 ± 0.65% difference at 100 nM (1.5 ng/mL, *p* < 0.005) Figure 5c. The PLIR for the Eu^3+^ chelated to PMDA modified anti-human myoglobin Affimer remained >1 (1.78–1.6) confirming that the Eu^3+^ fluorescence changes are also related to the ligand complex in a similar fashion to what was observed in Eu^3+^ chelated to PMDA modified anti-GFAP Affimer complex (Figure 4d).

### 3.5. Time-Resolved Fluorescence Intensity of Chelated Eu^3+^ to PMDA Modified Anti-CRP and Anti-CEA Affimer

**Figure 6:**
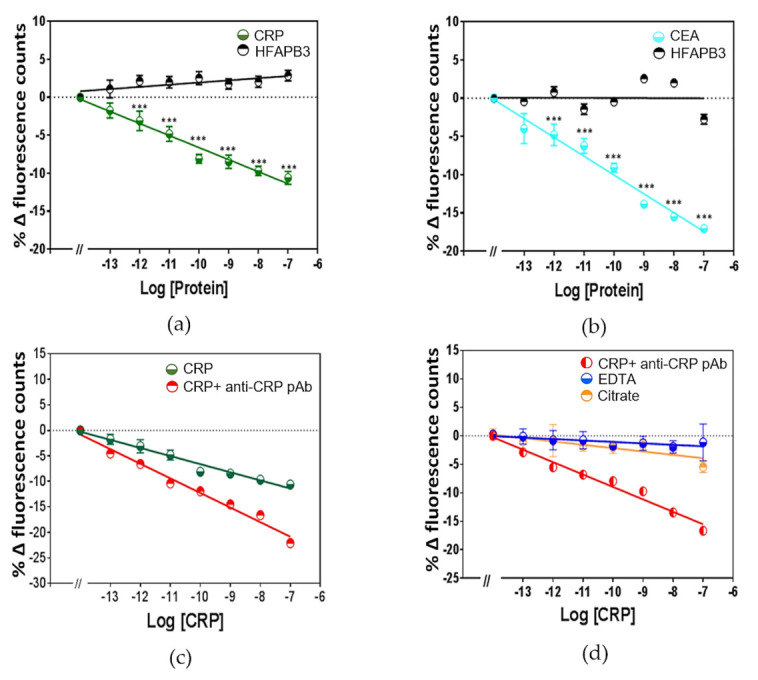
Time-resolved fluorescence counts of Eu^3+^ complex in 1% (*v*/*v*) serum. (**a**), time resolved fluorescence counts of Eu^+3^ chelated by PMDA modified anti-CRP Affimer; (**b**), time resolved fluorescence counts of Eu^+3^ chelated by PMDA modified anti-CEA Affimer. HFABP3 were used as a control. (**c**), time resolved fluorescence counts of Eu^3+^ complexes quenching against increased concentration of CRP from 100 fM to 100 nM whereas further quenching was observed following the addition of 100 nM polyclonal anti- CRP IgG. (**d**), time resolved fluorescence counts of Eu^+3^ chelated by PMDA modified anti-CRP Affimer followed adding of 10 µM EDTA and 1 µM citrate. Some error bars are smaller than data points (*, ** and *** indicate significance with *p*-value < 0.05, 0.01, and 0.001 respectively), (Data are means ± SEM (n = 3)).

The fluorescence spectrum of Eu^3+^ chelated complex was also investigated using time-resolved fluorescence measurement. Different analytes which are of different sizes were tested; CRP and CEA which are ~119 kDa and ~200 kDa, respectively. An integration start time (20 µs) and the measurement integration time of 50 s were chosen. In order to confirm accurate fluorescence intensity comparison between the fluorimeter measurement and the plate reader, the change in fluorescence intensity is presented using normalised data analysis. In Eu^3+^-chelated PMDA modified anti-CRP and PMDA modified anti-CEA Affimer complex (Figure 6a,b), substantial fluorescence quenching was observed as shown in both graphs. The fluorescence difference reached a statistical significance when Eu^3+^ complex was chelated by PMDA modified anti-CRP or PMDA modified anti-CEA Affimers at 100 fM (*p* < 0.005). The quenching of fluorescence intensity counts was directly proportional with increased concentration of their respective analytes from 100 fM to 100 nM in 1% (*v*/*v*) serum. Fluorescence quenching was further potentiated when compared to control analytes all of which is further confirmation of analyte size effect on Eu^3+^ complex fluorescence quenching. Nonetheless, the trend in fluorescence quenching was identical when compared to the results from Figure 4 and Figure 5. 

Time resolved fluorescence of the Eu^3+^ complex was further investigated to ascertain the size effect of the biomarker on the detection sensitivity. In order to increase the size of the biomarker, we performed a sandwich assay where the biomarker was bound by the Eu^+3^-Affimer complex and by an IgG simultaneously. The sensitivity of the Eu^3+^ complex biosensor was further enhanced when the analyte was simultaneously targeted using a sandwich format which has the Eu^3+^-anti-CRP Affimer complex and anti-CRP antibody both binding to CRP. The size effect of the antibody allowed significant increase in fluorescence quenching by 2-fold at 100 nM concentration; (Figure 6c).

### 3.6. Time-Resolved Fluorescence Intensity of Chelated Eu^3+^ to PMDA Modified Anti-CRP Affimer Complex with EDTA or Citrate

When considering the applicability of the Eu^3+^ complex for clinical use, attention has to be paid to the fact that blood samples are after collected in tubes EDTA or citrate present to prevent blood clotting. Accordingly, we assessed if chelators such as EDTA, citrate were confounding factors for any readings generated using the Eu^3+^ complex. When adding EDTA and citrate to the serum, no significant fluorescence quenching in Eu^3+^ chelated to PMDA modified anti-CRP Affimer complex was seen (Figure 6d). A potential explanation for the negative results we have seen is that, EDTA and citrate chelate the free Eu^3+^ in the serum.

## 4. Discussion

The data presented in this paper are the first to suggest a specific Eu^3+^ complex for the purpose of optical biosensing of human analytes using an Affimer as a bioreceptor. Using a biochemical approach, whereby Affimer scaffold lysine residues were modified to introduce three carboxy groups able to effectively chelate the Eu^3+^. The Eu^3+^ was excited at 395 nm while the emission wavelengths were 590 and 615 nm as shown in Figure 3. As the Eu^3+^ complex was tested in human serum the system was able to detect as low as 100 fM of the analyte’s concentrations but the system would only tolerate up to 1–2% (*v*/*v*) human serum. The percentage change in Eu^3+^ complex fluorescence when incubated with GFAP and human myoglobin (Figure 4 and Figure 5) confirmed the high sensitivity of the system at detecting different analytes.

The challenge in developing a lanthanide complex for biosensing against a specific analyte is to produce a stable detection system with reliable binding properties between the bioreceptor on the complex and the target analyte. Eu^3+^ complexes were reported to be used as luminescent probes sensing H_2_S, triplet oxygen, H_2_O_2_ and nitric oxide [43,44,45,46,47]. A common theme between all these Eu^3+^ complexes was the activation of an irreversible transformation of the ligand. The Eu^3+^ complex detection mechanism of the target analyte was based on Eu^3+^ emission enhancement. Although, all of the complexes allowed an efficient change in fluorescence response, the systems were of limited use. The main disadvantage of the systems was irreversibility. Accordingly, the design of such a system was useless for the purpose of our application where an accurate measurement of analyte concentration is important. Other interesting designs are probes for amino acids and protein detection [3,18,48,49,50,51,52,53], but selective detection of specific amino acids is very challenging because they share the same amine and carboxylic acid functional groups, Shinoda’s group (2014) were successful at establishing a combinatorial library to enhance lanthanide emission for selected amino acids. The library consisted of four different lanthanides (Eu^3+^, Tb^3+^, Nd^3+^, Yb^3+^), seven N-heteroaromatics and seven amino acid substrates to obtain 196 combinations [54]. Nonetheless, the responsive fluorescence of the complex was dependent primarily on the ligands employed and again did not allow specific concentration measurement. It would be almost impossible to detect analyte concentration using this system. The introduction of a protein targeting group to the ligand as an antenna enhanced luminescent lanthanide complexes for specific sensing of specific proteins. Guo’s group (2011), reported a Tb^3+^ complex with metronidazole as ligand, as the luminescent sensor for human serum albumin (HAS) [52]. They reported an increased emission intensity of Tb^3+^ upon addition of HAS. To date, there are no reports in the literature that describes a lanthanide complex system which allows specific analyte sensing and concentration measurements simultaneously. Our Eu^3+^ complex system appears to be the first to achieve this. The ligand in our system consists of PMDA modified lysine-NH_2_ groups acting as chelaters on an Affimer. Our hypothesis was that conjugating the ligand to the Affimer would aid establishing rapid and reliable system while the detection specificity would be enhanced via the bioreceptor recognition. Design of the experiments allowed appropriate use of control Affimer-Eu^3+^ complex and not just Eu^3+^ complex. This is a feature that allowed the quantification of fluorescence quenching to enable statistical comparison.

In Figure 6a,b we presented the results of the fluorescence measurements on Eu^3+^ chelated by PMDA modified Affimers against other higher Mr biomarker proteins, CRP and CEA using the plate reader as light energy source. Of clinical interest, the plate reader data showed that the Eu^3+^ complex allowed specific detection of the targeted analytes in as low as 100 fM concentration. Although the assay will only tolerate 1–2% (*v*/*v*) human serum, and so the real limit of detection (LOD) is around 100-fold higher, ~10 pM which is higher than the clinical cut-off of the tested biomarker. Importantly, the fluorescence quenching readings obtained from the plate reader were comparable to those observed when using the fluorimeter as reported in Figure 4 and Figure 5. Although, the measurement of the fluorescence quenching using the plate reader appeared to be slightly less sensitive when compared to the fluorimeter, the functionality of the plate reader allows many data replicates. This feature is essential for application in healthcare laboratories where large number of samples can be analysed simultaneously. As the biomarker was sandwiched between polyclonal antibody and Eu^3+^-Affimer complex (Figure 6c), the fluorescence quenching was further enhanced suggesting that the size of the analyte contributes substantially to the fluorescence quenching observed as shown in (Table 1). 

EDTA is a hexadentate ligand that contains four carboxylic acid groups and two amine groups [55]. It is able to chelate calcium and other metal ions [56]. The above features are exploited to prevent coagulation in vitro where binding to calcium, EDTA can prevent blood clotting and allows for whole blood analysis. EDTA is also used as a spray-coated to human blood sample collection tubes, at a concentration of 1–10 μM. Sodium citrate is another anticoagulant commonly used in blood collection tubes, with a 3.2% buffered sodium citrate solution and again woks by chelating calcium ions. The effects of EDTA and sodium citrate on the chelated Eu^3+^ complex is shown in Figure 6d and demonstrated that they interfere with the time-resolved fluorescence reading. This represents a potential challenge to the clinical application of Eu^3+^ complex unless the assay is carried out on human serum rather than plasma. Although, a potential solution to this limitation may be to use microfluidic sample handling. A lanthanide chelate based time-resolved fluorescence assay has been investigated by others and proved to be a successful for a wide range of areas, such as diagnostics, detection assays, microbes and biomarker discovery. Such a luminescent lanthanide complexes provide long-lasting fluorescence ranging from microseconds to milliseconds. The design of our Eu^3+^ complex experimental setting takes advantage of the above features and rely on the presence of the analyte to cause the quenching. 

The Affimer-based Eu^3+^ complexes showed wider detection range (100 fM–100 nM) when compared to other assays (Table 2). The sensitivity and detection range of ELISA and SPR followed in decreasing order. Importantly, the response time for the Affimer-based Eu^3+^ complexes was substantially shorter at 30 min (including calibration curve) when compared to ELISA (120–180 min) and SPR (6–24 h). Collectively, the Affimer-based Eu^3+^ complexes biosensor showed the most sensitive detection range and shortest response time. The advantage of our assay is that it is inexpensive (compared to SPR) and requires only a single reagent addition (as compared to ELISA). It also shows comparable or superior sensitivity.

## 5. Conclusions

In conclusion, we present a novel technique using an Affimer-based Eu^3+^-complex biosensor for the detection and measurement of a range of protein biomarkers in human serum. A range of analytes (CEA, CRP, GFAP and human myoglobin), which are different in their sizes (~200, 119, 55 and 17, respectively), were tested to ascertain the effect on the percentage change in fluorescence quenching of the Eu^3+^ complex. It is clear that the larger the analyte the higher fluorescence quenching was seen (Table 1). The biosensor showed a comparable or better sensitivity to ELISA for analyte detection but was much faster, since only addition of one solution is needed before measurement. The technology should be adaptable to target any specific analyte of clinical interest. The optimised sensors were highly sensitive with detection limit of <100 fM. The proteins which we tested using the Affimer based biosensor are commonly used as biomarkers in the clinic for diagnostic and prognostic purposes.

## Figures and Tables

**Table 1:** Showing the percentage quench in fluorescence intensity of the chelated Eu^3+^ to PMDA modified human myoglobin, GFAP, CRP and CEA Affimer in 1% (*v*/*v*) serum.

Biomarker	Molecular Weight (KDa)	% ∆ Fluorescence Counts of Biomarker	% ∆ Fluorescence Counts of Biomarker + Polyclonal Ab
Myoglobin	17	7.44 ± 1.17	17.25 ± 2.37
GFAP	55	4.66 ± 0.65	12.30 ± 0.46
CRP	119	10.05 ± 2.46	22.08 ± 0.85
CEA	200	17.05 ± 1.50	36.31 ± 6.24

∆ % fluorescence counts of chelated Eu^3+^ by PMDA modified human myoglobin, GFAP, CRP and CEA Affimers were measured at 100 nM concentration of biomarker. Data are means ± SEM (n = 3).

**Table 2:** Showing comparison between the Eu^3+^ chelated biosensor detection range and other biosensor assays.

Biomarker	Eu^3+^-Chelated Biosensor	ELISA	SPR	Reference
Myoglobin	100 fM–100 nM	10 pM–100 µM	3.6 ± 0.02 nM	[57]
GFAP	100 fM–100 nM	1 pM–1nM	35.8 ± 0.01 nM	[57]
CRP	100 fM–100 nM	>µM	>10 pM	[21,58]
CEA	100 fM–100 nM	10 pM	34.4 ± 16 nM	[22]
